# Bridging Self‐Assessment Gaps: The Impact of Objective Structured Clinical Examination (OSCE) on Nursing Students' Competency Perception: A Pre‐Post‐Test Study

**DOI:** 10.1002/nop2.70487

**Published:** 2026-04-30

**Authors:** Virginia La Rosa‐Salas, Carmen Fernández‐Panadero, Miriam Pereira‐Sánchez, Miren Idoia Pardavila‐Belio, Marta Lizarbe‐Chocarro

**Affiliations:** ^1^ Department for Community, Maternal and Child Nursing University of Navarra Pamplona Spain; ^2^ ICCP‐UNAV Research Group, Innovation for Person‐Centred Care University of Navarra Pamplona Spain; ^3^ Institute of Health Research of Navarra (IdiSNA) Pamplona Spain; ^4^ Departamento de Ingeniería Telemática University Carlos III from Madrid Leganés, Madrid Spain

**Keywords:** competence self‐assessment, nursing education, Objective Structured Clinical Examination, OSCE, self‐regulated learning, undergraduate nursing student

## Abstract

**Aim:**

Self‐assessment is critical in nursing education, enabling students to reflect on their cognitive and clinical skills while helping educators identify gaps in competencies. Combining subjective self‐assessment with the objectivity of Objective Structured Clinical Examinations (OSCEs) supports learning adjustments. This study explores the impact of OSCEs on nursing students' self‐assessment of practical competencies.

**Design:**

A quasi‐experimental pre‐post study was conducted with a convenience sample of 229 third‐ and fourth‐year nursing students at a Spanish university. Only students who had not previously taken an OSCE were included. A 10‐station OSCE was implemented, and the Self‐Assessment Competence Scale (SACS) evaluated perceived competence levels. Data analyses included descriptive statistics, sensitivity testing, *t*‐tests, and ANOVA (tertile analysis) with size effect for group differences, and regression analysis to detect the Dunning‐Kruger effect.

**Results:**

Fourth‐year students scored significantly higher on OSCEs than third‐year students. Female and male students had similar OSCE results, though fourth‐year students demonstrated higher self‐perception of competence than their third‐year peers. The mean of self‐assessment scores is similar for men and women, but disaggregating scores by competences shows significant differences. Interestingly, students with higher self‐perceived competence often achieved lower OSCE scores, revealing discrepancies attributed to the Dunning‐Kruger effect. Post‐OSCE, students adjusted their self‐perception more closely to their actual performance.

**Conclusion:**

OSCEs effectively identify and address discrepancies between self‐assessment and objective performance, fostering critical self‐awareness among nursing students. By revealing overconfidence in less skilled students, these findings highlight the importance of integrating structured OSCEs into nursing curricula. Educators can use these insights to develop targeted programs that enhance competency development, reflective learning and patient safety.

## 1 Introduction

The objective of transforming nursing education is to improve training in order to equip students with the requisite competencies to ensure safe, high‐quality patient care. A pivotal element of this educational process is assessing students' progress toward the established objectives that define nursing competencies (Alkhelaiwi et al. 2024; Kelly and Lazenby 2018; Lachmann and Nilsson 2021), and can be approached from two complementary perspectives: objective evaluation and self‐assessment. To assess the acquisition of these competencies through an objective evaluation, a growing number of nursing schools require students to pass a summative Objective Structured Clinical Examination (OSCE) (Llaguno et al. 2024; Solà‐Pola et al. 2020).

Summative OSCEs are important complements to nurse–educator evaluations for ensuring that all students have achieved mastery of clinical acumen as defined by a school's learning outcomes. That is why, in clinical nursing education, student evaluations often rely on subjective assessments by individual nurses, which can vary greatly and compromise the reliability of grading. This inconsistency poses a challenge in ensuring fair and accurate measurement of clinical competencies. Objective Structured Clinical Examinations (OSCEs) help overcome these issues by offering a standardised and objective method of evaluation, reducing assessor bias and ensuring all students are measured against the same criteria and scenarios (Yusuf 2021). This challenge is also evident in our context, where variability in clinical assessments has raised concerns regarding consistency and fairness.

Moreover, a critical component of clinical acumen is the ability to recognise one's own limitations, particularly in terms of knowledge and skills (Newton and Smith 2024), and to identify one's own gaps and needs for competence development (Forsman et al. 2020). The OSCE provides a structured yet realistic clinical environment that closely mirrors real‐life practice, making it a suitable setting for students to reflect on their own performance. Its standardised format enables students to evaluate their abilities against transparent criteria, supporting accurate and meaningful self‐assessment and reducing the risk of developing a false perception of their competence. This concern is also evident in our own context, where students' difficulties in accurately assessing their clinical abilities have been observed during recent evaluation processes.

Given this background, it becomes essential to explore how OSCEs may contribute to fostering more accurate self‐assessment in our setting.

## 2 Background

Self‐assessment is particularly important for nursing students, as it allows for reflection and evaluation of their advanced cognitive and clinical skills. This process is equally vital for educators, allowing the identification of knowledge and skill gaps that facilitate the development of competencies that are aligned with professional standards for complex patient care (Immonen et al. 2019; Jessee 2021). Defined as “the act of monitoring one's processes and products in order to make adjustments that deepen learning and enhance performance” (Andrade 2019), self‐assessment involves ongoing subjective evaluation and is closely related to self‐monitoring and reflective practices (Yan 2020). Students' abilities to evaluate both their own and others' work are essential for ensuring patient safety and achieving professional competence (Henderson et al. 2022).

Moreover, self‐assessment enhances learners' self‐responsibility and self‐regulation in learning (SRL), empowering them to establish autonomy and control over their performance (Piper et al. 2019; To and Panadero 2019). Effective self‐assessment is crucial for ongoing professional development, with evidence suggesting that self‐directed feedback seeking during performance phases predicts academic success (Yan 2020). However, discrepancies between subjective self‐assessment and actual performance can threaten patient safety (Knof et al. 2024), as previous studies have shown contradictions between self‐assessments and evaluations by qualified nurses and mentors (Kajander‐Unkuri et al. 2020).

Objective structured clinical examinations offer a structured assessment approach that may help mitigate these discrepancies (Yusuf 2021). This comprehensive evaluation method, which combines global rating scales with checklists, has been used in nursing education for decades to gauge clinical competence and readiness for practice (Halman et al. 2020; Lee et al. 2019). OSCEs, which have been validated as reliable tools (Lee et al. 2019), assess cognitive, affective and psychomotor domains, as aligned with the “shows how” level of the Miller pyramid (Miller 1990). By employing task analyses specific to nursing programs, OSCEs typically occur in simulated environments, where essential competencies are evaluated through structured stations (Lee et al. 2019).

Despite the necessity of combining self‐assessment with objective examiner evaluations for effective nursing education, studies have revealed little correlation between self‐assessment and objectively measured competence (Høegh‐Larsen et al. 2023; Immonen et al. 2019). Furthermore, the findings indicate that students who perform poorly on self‐assessment tend to overestimate their abilities, whereas those who excel tend to assess themselves more accurately or even underestimate their performance (Høegh‐Larsen et al. 2023; Rahmani 2020). This discrepancy is often attributed to the educational psychological phenomenon identified as the Dunning–Kruger effect (Kruger and Dunning 2000), wherein individuals with lower competence tend to overestimate their abilities. It impairs an individual's capacity for decision‐making, particularly in the context of academic pursuits such as studying and self‐directed learning, and consequently, it hinders the learning curve of each student. Such misjudgments can lead to inaccurate evaluations in educational outcomes, potentially compromising patient safety (Høegh‐Larsen et al. 2023; Kruger and Dunning 2000). Prior research has focused primarily on the practical skills of medical (Knof et al. 2024) and nursing students (Høegh‐Larsen et al. 2023).

To date, five studies have examined the relationship between self‐assessed competence levels and performance evaluations for nursing students. Two studies specifically addressed emergency situations and anaesthesia (Jeon et al. 2020). However, general nursing competencies remain underexplored, as researchers assert that bachelor's‐level education often lacks sufficient training for emergency nursing. One study compared self‐assessments of clinical judgement in both simulation and clinical practice but did not focus solely on OSCEs (Høegh‐Larsen et al. 2023). The remaining studies employed cross‐sectional designs, with Fekonja et al. (2017) assessing self‐assessment post‐OSCE without pre‐OSCE data, whereas Kajander‐Unkuri et al. (2020) evaluated self‐assessment congruence with mentor assessments after clinical placements, excluding OSCE data (Fekonja et al. 2017; Kajander‐Unkuri et al. 2020).

Thus, the aim of this study was to examine the influence of OSCEs on the self‐assessment of undergraduate nursing students. Specifically:

1. Explore the influence of OSCEs on the self‐assessment of general practical nursing competencies using a pre‐post‐test design.
2. Investigate the association between self‐assessment of the general practical competencies of nursing students and their OSCE grades.
3. Examine the presence of the Dunning–Kruger effect among nursing students' self‐assessed competence levels.

## 3 Methods

### 3.1 Design

A quasi‐experimental pre‐post‐test study was conducted at a Spanish university to evaluate the effect of an OSCE on the self‐assessment of nurses' general competences. This study was reported in accordance with the Reporting Guidelines for Health Care Simulation Research: extension to the CONSORT and STROBE statements.

### 3.2 Research Setting

The research was undertaken at the Simulation Centre of the Faculty of Nursing at the University of Navarra, located in northern Spain.

### 3.3 Participants and Recruitment

The target group for the study consisted of third (*n* = 117) and fourth (*n* = 132) year nursing students who met as inclusion criteria to have completed theoretical courses in physiology, anatomy, pharmacology, pathology and core nursing issues related to chronic and acute care. Participants were also required to have successfully finished at least three rotating internships: a six‐week and a 10‐week clinical placement in a general hospital and a 6‐week placement in a primary care setting. Furthermore, attendance at required practical nursing skills classes and participation in eight simulation sessions were prerequisites. Students who had previously completed an OSCE were excluded from the study.

Recruitment for the study took place in spring 2019 and involved disseminating information during the Practicum III (pre‐final year) and Practicum V (final year) courses by the research team. Eligible students were informed about the study's aims, data collection methods, confidentiality measures, voluntary participation and their right to withdraw at any time.

**FIGURE 1:**
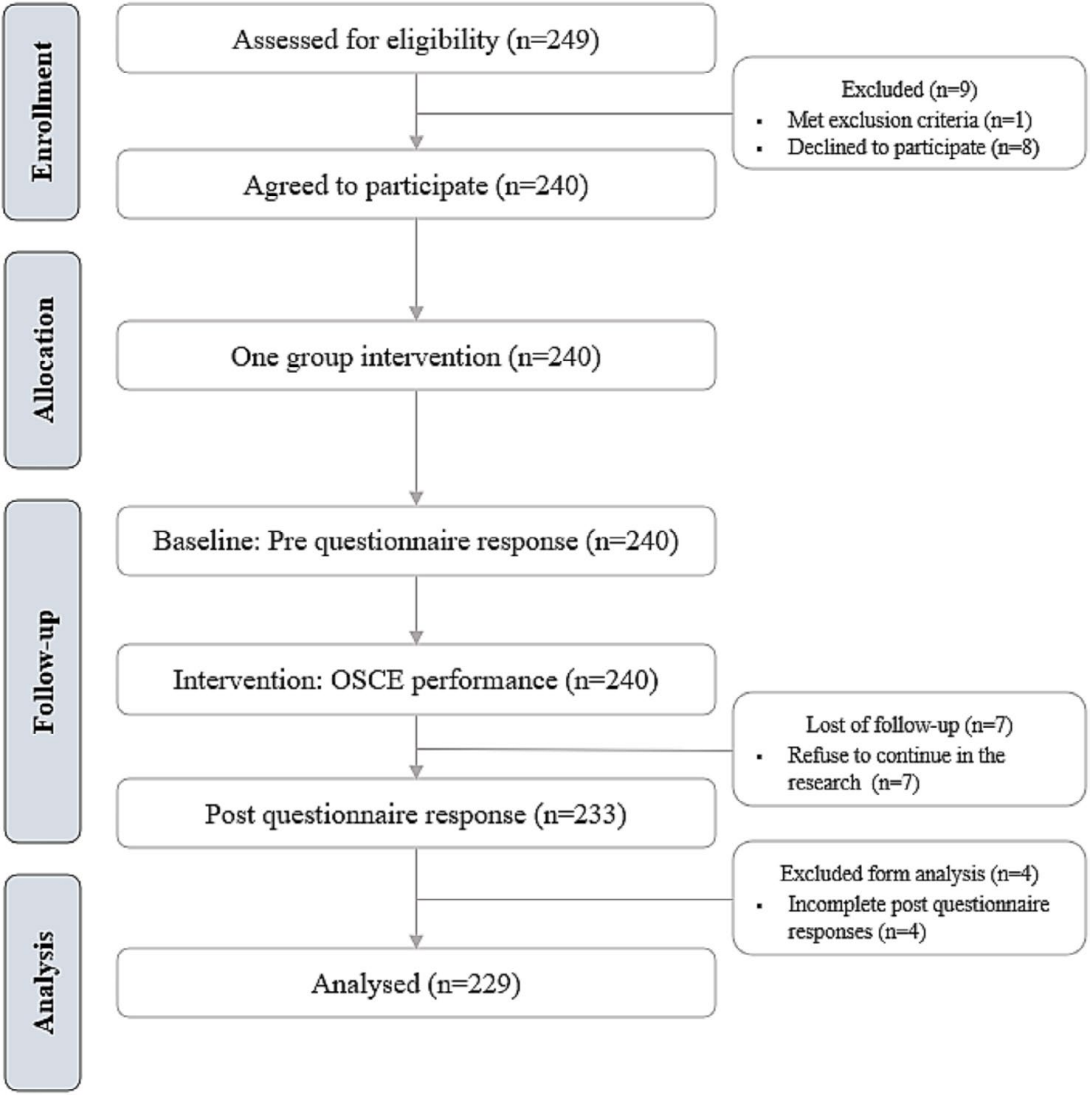
Consolidated Standards of Reporting Trials, CONSORT, flowchart of participants throughout the pre‐post study.

A total of 249 students were invited to participate in this study. One student was excluded for being a repeat participant who had attempted the OSCE the previous year, and eight declined to participate. Additionally, 11 other students (4.6% attrition) were excluded because they refused to continue with the research and because their post‐questionnaire data were incomplete. Thus, the final sample for the study included 229 students (Figure 1).

### 3.4 Intervention

#### 3.4.1 Objective Structured Clinical Evaluation (OSCE)

To design and prepare the OSCE, the course objectives, contents and clinical skills requirements were thoroughly reviewed. Moreover, assessment content was meticulously planned on the basis of the learning objectives (blueprinting). To ensure the OSCE's face and content validity, all stations were developed by an expert panel. This expert panel was composed of five registered nurses (RNs) and four lecturers from the School of Nursing specialising in competence assessment and simulation. The RNs had at least 5 years of clinical experience in different clinical services, such as emergency services or postsurgical hospitalisation units, among others. The faculty lecturers had at least 3 years of simulation experience and had deep knowledge of the creation and use of evaluative rubrics; one was also a specialist in mental health and behavioural changes, and the other was a specialist in the surgery field. The expert panel developed 10 OSCE stations that incorporated 10 professional core competencies from the nursing curriculum (Table 1).

**TABLE 1:** OSCE stations description and competence loads.

OSCE stations	Competence loads (%)										
	C_1_	C_2_	C_3_	C_4_	C_5_	C_6_	C_7_	C_8_	C_9_	C_10_	Total (%)
primary care	10			60	20			10			100
Paediatric intervention	20		10		10	20	20	10		10	100
Aspiration of respiratory secretions & oxygen therapy	40				20		20	20			100
Medication administration	50						30	20			100
Septic patient care	40				10	20	20	10			100
Bladder catheterization	40				10	10	10	10	20		100
Venous access	40						30	30			100
Knowledge test	10	10	10	10	10	10	10	10	10	10	100
Clinical material identification	20		10		20		20	20			100
Reflective journal	10	10	10	10	10	10	10	10	10	10	100
Total	280	20	40	80	110	70	170	150	40	20	

Abbreviations: C_1_, Nursing process; C_10_, Ethical decision making; C_2_, Palliative care; C_3_, Collection and registration's information; C_4_, Health promotion and educational interventions; C_5_, Therapeutic relationship; C_6_, Team working; C_7_, Safety health care; C_8_, Reflective learning; C_9_, Patients values and believes; OSCE, objective structured clinical examination.

The evaluation of each station was carried out by an examiner using a specifically designed checklist with a rating form based on a rubric evaluation. The total score of the checklist items ranged from 0 to 1; each item was graded on a 3‐point scale including “complete success” (0.1 points), “partial success” (0.05 points) and “complete failure” (0 points), with scores of up to 10 points overall on the OSCE. The scoring system classified performance into four levels: scores below 5 indicated that the student had not met the minimum required level of competence; scores from 5 to 6.9 reflected achievement of the minimum competence level; scores from 7 to 8.9 indicated a notable level of competence; and scores of 9 or above represented excellent competence. This categorization offers a clear and objective framework for interpreting OSCE scores and assessing students' clinical readiness.

An illustrative example is provided below, based on a paediatric cardiopulmonar resuscitation (CPR) station, to clarify how clinical performance was assessed using the OSCE rubric. This station assessed students' ability to deliver high‐quality basic life support in a simulated scenario. The assessment rubric for this station included the following criteria: (1) Initial Assessment and Call for Help: Prompt and appropriate response to the situation; Assessment of responsiveness (e.g., “unresponsive” status); Immediate activation of emergency medical services or call for help; Simultaneous assessment for normal breathing or signs of life (e.g., crying or coughing in infants). (2) Airway Management and Ventilation: Correct airway opening technique, including a neutral head position for infants under 1 year; Delivery of five initial rescue breaths. Accurate mouth‐to‐nose ventilation technique. (3) Chest Compressions: Initiation of high‐quality (e.g., adequate depth, rate and recoil); chest compressions following the initial breaths; Correct fingertip technique for infant compressions; Maintenance of the appropriate compression‐to‐ventilation ratio (15:2) for two‐rescuer infant/child CPR. (4) Algorithm Completion: Activation of the resuscitation or Advanced Life Support (ALS) team after 1 min of continuous CPR.

To ensure the rigour of the OSCE, the same examiner observed and filled out the checklist for all students' performance at the same station. The examiners were both RNs with more than 10 years of clinical experience (*N* = 8), and two faculty lecturers with more than 15 years of nursing teaching experience. All of them received 3 h of training delivered by the principal investigator, who chaired the OSCE Committee.

### 3.5 Instruments

#### 3.5.1 Self‐Assessment Competence Scale (SACs)

To evaluate nursing students' self‐assessment of their competence levels, the Self‐Assessment Competence Scale (SACs) was employed. The instrument was developed in accordance with the Spanish Ministerial Order regulating official university nursing degrees, ensuring national relevance and alignment (Rodríguez‐Higuera 2014). It was specifically designed to assess undergraduate nursing students' perceived competence prior to undertaking an OSCE. Findings from an initial sample of 46 students were benchmarked against objective OSCE results, showing good face and content validity (Rodríguez‐Higuera 2014).

Comprising 14 items distributed across seven domains, this scale evaluates self‐perceived competence in nursing students. The domains include information collection and documentation (items 1 & 2), clinical assessment (items 3 & 4), nursing skills (items 5, 6 & 8), ethical decision‐making (item 7), therapeutic relationships (items 9 & 10), health promotion and educational interventions (items 11 & 12), and teamwork (items 13 & 14). Each item is rated on a 10‐point Likert scale, from 0 (never) to 10 (always), resulting in a total score between 0 and 140 points. Although no formal cut‐off has been established, higher scores reflect greater levels of self‐perceived competence.

In the present study, Cronbach's alpha coefficient for the SACs was 0.91. Corrected item–total correlations for all items were above 0.40, and removal of any single item did not increase the alpha coefficient. These findings indicate that each item contributes meaningfully to the overall scale, demonstrating that the SACs is a reliable instrument for assessing self‐perceived competence in nursing students.

### 3.6 Study Procedures

**FIGURE 2:**
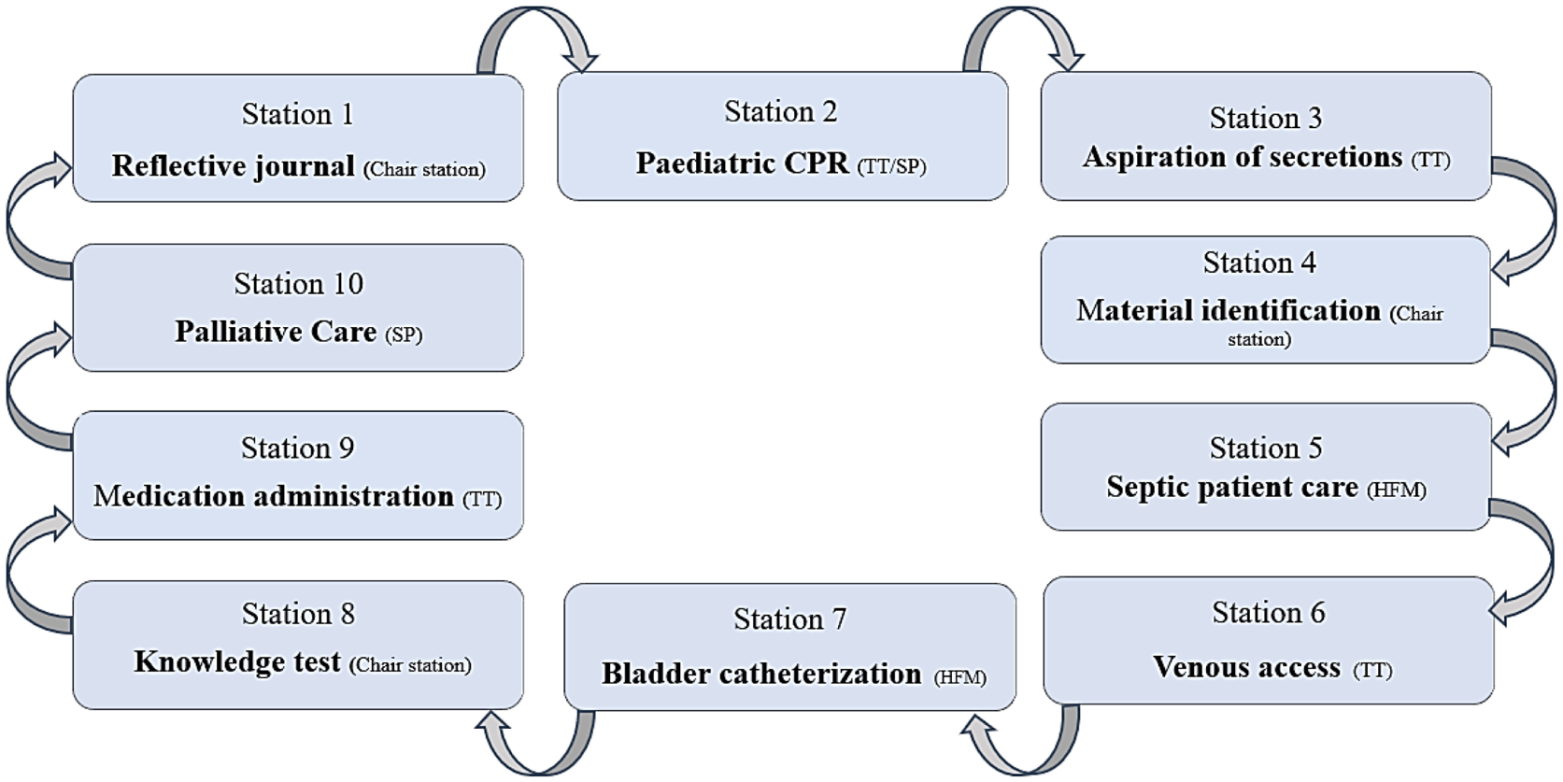
OSCE stations wheel. CPR, cardiopulmonary resuscitation; HFS, high fidelity simulator; SP, standardized patient; TT, task trainer.

Prior to the OSCE, all students completed a written SACs questionnaire (with a mean duration of 7 min). After that, students proceeded to undertake the OSCE, which consisted of 10 examiner observation stations. The duration of the stations was 8 min; however, for some students with special capabilities, more time was required (an increase of 25% was applied to each station). The transition time between stations was 2 min. Each OSCE station was set up in a similar fashion and included all the necessary equipment (task trainer, high‐fidelity mannequin or standardised patient) and resources that the student would need to resolve each clinical case (Figure 2). Once the OSCE had been completed, the students proceeded to undertake the SACs once more, this time without being aware of their OSCE marks.

### 3.7 Data Analyses

Categorical variables were presented as frequencies and percentages, and continuous variables as central tendency measures (means, medians, standard deviations‐SD and ranges). A sensitivity analysis by course and gender was carried out. Student's *t*‐test was used to identify differences between groups, and the paired sample *t*‐test was used to study the SACs at two different time points, i.e., the pre‐ and post‐intervention results. To assess the effect size, Cohen's *d* was calculated in accordance with the guidelines for the design of pre‐post‐test studies (Cohen 1988). A result of less than 0.2 was defined as a weak effect size; from 0.2 to 0.49 was defined as a small effect size; from 0.5 to 0.79, as a medium effect size; and more than 0.8 as a large effect size (Cohen 1988). To investigate the relationships between OSCE and the pre‐ and post‐OSCE SACs results, the Pearson correlation coefficient was used. The level of correlation was interpreted as weak (< 0.30), moderate (0.30–0.69) or strong (> 0.70) (Hemphill 2003). Bland–Altman plots were generated to illustrate the average bias and to test whether there were systematic differences between self‐assessment students' competence and OSCE competence evaluation. Linear regression analysis and a scatter plot were used to study the relationships among variables and determine whether the Dunning–Kruger effect was present in nursing students' self‐assessment of their competence levels through the difference between externally‐evaluated objective student competency level (determined by the OSCE) and internally‐evaluated subjective student self‐assessed competency level (assessed through SACs‐PRE variables). To delve deeper into this relationship, a sensitivity analysis by gender and academic year, by tertiles combined inter‐group one‐way ANOVA test. All data analysis was performed using the statistical software package SPSS version 2.0 (SPSS Inc., Chicago, USA), and the results were considered statistically significant at < 0.05 on a two‐tailed basis for all tests.

### 3.8 Ethical Consideration

The research protocol was conducted under the guidelines established by the University of Navarra for educational innovation studies. Specifically, the study was approved by the head of the nursing faculty and the directors of the relevant course sections. Participants were provided with a detailed information sheet and informed consent form, clearly outlining the research objectives, methods and the intention to publish results while ensuring confidentiality. This information was conveyed both orally and in writing to clarify and transparency. The OSCE was conducted as a mandatory class activity; however, participation in the pre‐ and post‐test surveys was voluntary and students were informed of their right to withdraw at any point without academic repercussions. Additionally, data was securely stored on university servers and will remain so for a period of 5 years, in compliance with institutional policies and data protection regulations. These measures ensured adherence to ethical and scientific rigour while prioritising participants' rights and data integrity.

## 4 Results

### 4.1 Demographic Data

The 229 participants in the study had a mean age of 21.5 years (SD = 1.6). Additionally, 197 identified as female (86%). Regarding the distribution by year in the Nursing Bachelor Program, 43.7% were in the third year and 56.3% were in the fourth year.

### 4.2 Description of the OSCE

The mean overall OSCE score was 7.54 (SD = 0.6; range = 5.65–9.15). While female students presented slightly higher scores than their male counterparts did (7.56 for women and 7.39 for men), the difference was not statistically significant. This was a small effect size, with Cohen's *d* of 0.24. Moreover, students in their fourth year had an average OSCE score of 7.64 points, which was significantly higher than that of students in their third year (7.42 points) (*t* = −2.40; *p* = 0.017). This was a small effect size, with Cohen's *d* of 0.31.

### 4.3 Pre‐Test and Post‐Test SACs Results

Before the OSCE, the total overall SACs score was 111.6 out of 140. Women had slightly higher total SAC scores than men did, and students in the fourth year of degree had significantly higher scores than those in the third year (113.6 and 109.6, respectively; *t* = −3.26, *p* = 0.001). After the OSCE, the total SACs score was 112.2, which was similar for females and males but higher for students in the fourth year than for students in the third year (114.9 and 108.7 points, respectively; *t* = −3.97, *p* < 0.001) (Tables 2 and 3).

**TABLE 2:** Pre‐test and post‐test Self‐Assessment Competence scale (SACs) by gender.

Female (*n* = 197)	Pre‐test	Post‐test	*t* value	*p* *
	Mean (SD)	Mean (SD)		
Item1. Collect the information to determine the patient's health status or situation	7.85 (0.99)	7.68 (1.23)	2.09	0.038
Item2. Write down the patient information clearly	7.91 (1.16)	7.69 (1.24)	2.41	0.017
Item3. Value their symptoms and signs to specify the patient's problem	7.63 (1.06)	7.64 (1.14)	−0.12	0.907
Item4. Define the real and/or potential problems using nursing diagnoses	7.19 (1.15)	7.40 (1.20)	−2.70	0.007
Item5. Perform nursing procedures (skills)	7.95 (1.04)	7.71 (1.25)	3.08	0.002
Item6. Show skill in the procedures	7.73 (1.09)	7.51 (1.28)	2.83	0.005
Item7. Adjust the decisions and behaviours to the bioethical principles	8.77 (1.15)	8.87 (1.14)	−1.26	0.209
Item8. Have in mind at all times the theoretical knowledge for the development of practical ability	7.63 (1.09)	7.73 (1.23)	−1.27	0.204
Item9. Establish good communication	8.61 (1.05)	8.53 (1.15)	1.03	0.303
Item10. Create a relationship of empathy	9.01 (0.98)	8.92 (1.01)	1.47	0.144
Item11. Educate the patient in the prevention of health problems	7.86 (1.16)	8.16 (1.08)	−3.79	< 0.001
Item12. Promote actions that lead to change of habits and the promotion of health	7.83 (1.11)	8.17 (1.06)	−4.50	< 0.001
Item13. Lead the action within the working group	7.24 (1.25)	7.52 (1.36)	−3.62	< 0.001
Item14. Integrate and make other colleagues participate in teamwork	8.70 (1.10)	8.64 (1.21)	0.55	0.584
Total SACs	111.90 (10.89)	112.20 (12.46)	0.40	0.690

Male (*n* = 32)	Pre‐test	Post‐test	*t* value	*p* *
	Mean (SD)	Mean (SD)		
Item1. Collect the information to determine the patient's health status or situation	7.63 (1.16)	7.75 (0.95)	−0.68	0.501
Item2. Write down the patient information clearly	7.91 (1.01)	7.66 (1.12)	1.44	0.161
Item3. Value their symptoms and signs to specify the patient's problem	7.69 (0.93)	7.88 (0.91)	−1.53	0.136
Item4. Define the real and/or potential problems using nursing diagnoses	7.28 (1.11)	7.56 (0.95)	−1.79	0.083
Item5. Perform nursing procedures (skills)	8.38 (1.07)	8.38 (0.98)	0.00	1.000
Item6. Show skill in the procedures	8.22 (0.97)	8.06 (1.13)	0.84	0.407
Item7. Adjust the decisions and behaviours to the bioethical principles	8.22 (1.16)	8.25 (1.05)	−0.15	0.879
Item8. Have in mind at all times the theoretical knowledge for the development of practical ability	7.78 (1.09)	7.66 (1.23)	0.64	0.525
Item9. Establish good communication.	8.19 (1.14)	8.38 (1.16)	−1.03	0.311
Item10. Create a relationship of empathy	8.56 (1.07)	8.56 (1.22)	0.00	1.000
Item11. Educate the patient in the prevention of health problems	7.93 (1.13)	8.31 (1.09)	−1.83	0.076
Item12. Promote actions that lead to change of habits and the promotion of health	7.78 (1.09)	8.13 (1.16)	−1.77	0.086
Item13. Lead the action within the working group	7.28 (1.02)	7.59 (1.10)	−1.62	0.115
Item14. Integrate and make other colleagues participate in teamwork	8.09 (1.11)	8.00 (1.16)	0.42	0.675
Total SACs	110.93 (10.47)	112.16 (10.87)	0.96	0.345

Abbreviation: SD, standard deviation.

* Student's paired test.

**TABLE 3:** Pre‐test and post‐test Self‐Assessment Competence scale (SACs) by year of degree.

Third course (*n* = 100)	Pre‐test	Post‐test	*t* value	*p* *
	Mean (SD)	Mean (SD)		
Item1. Collect the information to determine the patient's health status or situation	7.61 (1.07)	7.45 (1.21)	1.25	0.213
Item2. Write down the patient information clearly	7.73 (1.20)	7.45 (1.23)	2.32	0.022
Item3. Value their symptoms and signs to specify the patient's problem	7.42 (1.08)	7.44 (1.13)	−0.16	0.870
Item4. Define the real and/or potential problems using nursing diagnoses	7.02 (1.15)	7.16 (1.06)	−1.37	0.175
Item5. Perform nursing procedures (skills)	7.62 (1.08)	7.41 (1.39)	1.75	0.083
Item6. Show skill in the procedures	7.46 (1.11)	7.18 (1.40)	2.35	0.021
Item7. Adjust the decisions and behaviours to the bioethical principles	8.56 (1.23)	8.61 (1.16)	−0.39	0.694
Item8. Have in mind at all times the theoretical knowledge for the development of practical ability	7.38 (1.05)	7.48 (1.29)	−0.86	0.394
Item9. Establish good communication	8.48 (1.08)	8.36 (1.21)	1.10	0.275
Item10. Create a relationship of empathy	8.83 (1.00)	8.75 (1.11)	0.80	0.426
Item11. Educate the patient in the prevention of health problems	7.77 (1.25)	7.88 (1.10)	−0.97	0.335
Item12. Promote actions that lead to change of habits and the promotion of health	7.80 (1.17)	7.93 (1.02)	−1.23	0.223
Item13. Lead the action within the working group	7.02 (1.14)	7.22 (1.32)	−1.69	0.093
Item14. Integrate and make other colleagues participate in teamwork	8.46 (1.20)	8.27 (1.19)	1.41	0.161
Item Total SACs	109.16 (11.00)	108.59 (12.28)	0.62	0.535

Fourth course (*n* = 129)	Pre‐test	Post‐test	*t* value	*p* *
	Mean (SD)	Mean (SD)		
Item1. Collect the information to determine the patient's health status or situation	7.98 (0.95)	7.87 (1.14)	1.21	0.230
Item2. Write down the patient information clearly	8.05 (1.06)	7.87 (1.19)	1.62	0.108
Item3. Value their symptoms and signs to specify the patient's problem	7.80 (0.99)	7.85 (1.08)	−0.47	0.637
Item4. Define the real and/or potential problems using nursing diagnoses	7.35 (1.12)	7.61 (1.20)	−3.00	0.003
Item5. Perform nursing procedures (skills)	8.31 (0.93)	8.10 (1.02)	2.29	0.023
Item6. Show skill in the procedures	8.06 (0.97)	7.90 (1.07)	1.81	0.073
Item7. Adjust the decisions and behaviours to the bioethical principles	8.00 (1.11)	8.92 (1.12)	−1.39	0.168
Item8. Have in mind at all times the theoretical knowledge for the development of practical ability	7.86 (1.07)	7.90 (1.15)	−0.45	0.656
Item9. Establish good communication	8.60 (1.07)	8.63 (1.08)	−0.27	0.790
Item10. Create a relationship of empathy	9.04 (0.99)	8.97 (1.00)	1.08	0.280
Item11. Educate the patient in the prevention of health problems	7.95 (1.07)	8.41 (1.01)	−4.91	< 0.001
Item12. Promote actions that lead to change of habits and the promotion of health	7.84 (1.06)	8.34 (1.08)	−5.54	< 0.001
Item13. Lead the action within the working group	7.42 (1.18)	7.77 (1.28)	−3.97	< 0.001
Item14. Integrate and make other colleagues participate in teamwork	8.72 (1.04)	8.78 (1.12)	−0.60	0.549
Total SACs	113.79 (10.23)	114.91 (11.51)	−1.60	0.112

Abbreviation: SD, standard deviation.

* Student's paired test.

No significant differences according to gender or course were found between the total pre‐ and posttest SACs results, with a weak effect size and a Cohen's *d* of 0.11 and 0.10, respectively. However, after the scale items were grouped by competence clusters, the OSCE decreased the self‐assessment perceptions of the competence group with respect to *Collection and registration information* (*t* = 2.61; *p* = 0.010), *Nursing skills* and *Therapeutic relationship* (*t* = 2.07; *p* = 0.039). In contrast, the scores for the competence group *Clinical Assessment* (*t* = −2.05; *p* = 0.041) and *Health promotion* and *Education interventions* (*t* = −4.93; *p* < 0.001) increased significantly from the pre‐ to posttest timepoints (Table 4). This was a weak effect size, with Cohen's *d* of 0.14.

**TABLE 4:** Pre‐test and post‐test Self‐Assessment Competence Scale (SACs) by competence group.

	SACs pre‐test	SACs post‐test	*t* *	*p*
	Mean (SD)	Mean (SD)		
Collection and registration's information	15.70 (1.90)	15.40 (2.23)	2.61	0.010
Clinical assessment	14.83 (2.97)	15.10 (2.08)	−2.05	0.041
Nursing skills	23.44 (2.74)	23.10 (3.30)	2.07	0.039
Ethical decision making	8.74 (1.17)	8.80 (1.15)	−1.00	0.221
Therapeutically relationship	17.54 (1.91)	17.40 (2.07)	−1.05	0.291
Health promotion and education interventions	15.74 (2.16)	16.30 (2.06)	−4.93	< 0.001
Team working	15.90 (2.00)	16.10 (2.24)	−1.86	0.064

*Note:* Collection and registration's information: items 1 & 2; Clinical assessment: items 3 & 4; Nursing skills: items 5, 6 & 8; Ethical decision making: item 7; Therapeutically relationship: items 9 & 10; Health promotion and education interventions: items 11 & 12; Team working: items 13 & 14.

Abbreviation: SD, standard deviation.

* *t* student paired data.

### 4.4 Relations Among the OSCE, Pretest SACs Scores and Posttest SACs Scores

**FIGURE 3:**
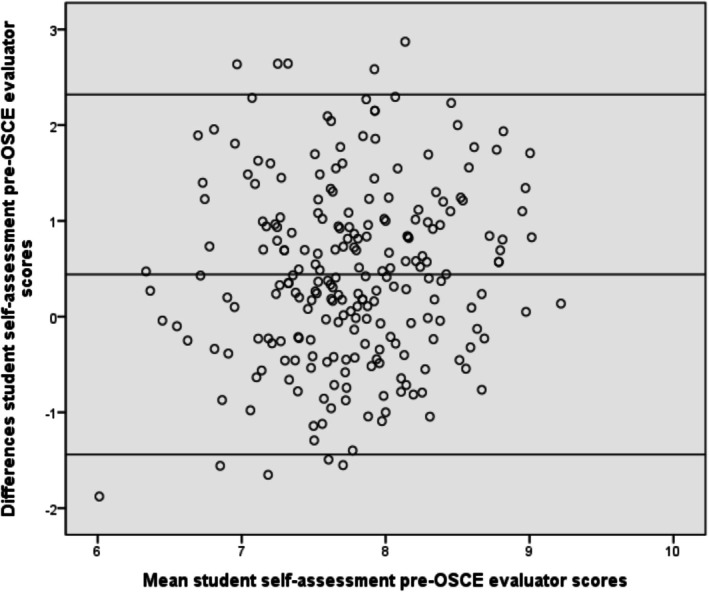
Bland–Altman plots of student competences self‐assessment before the OSCE and OSCE competence evaluation.

The OSCE scores had a weak correlation with the pretest SACs scores (Pearson coefficient = 0.185; *p* < 0.050) and a moderate correlation with the posttest SACs scores (Pearson coefficient = 0.351; *p* < 0.010) (Table 5). As we can see in Figure 3, the Bland–Altman plots show a systematic difference and wide limits of agreement between students' competence self‐assessment (subjective self‐assessment) and OSCE competence evaluation (objective).

**TABLE 5:** Correlations (Person's coefficient) between OSCE, SACs‐pre and SACs‐post‐test.

	OSCE	SACs pre‐test	SACs post‐test
OSCE	1	0.185*	0.351**
SACs pre‐test	0.185*	1	0.732**
SACs post‐test	0.351**	0.732**	1

*Note:* Pearson's correlation coefficient. The level of correlation was interpreted according to Cohen (2013) as weak (< 0.30), moderate (0.30–0.69), or strong (> 0.70).

* *p* < 0.05.

** *p* < 0.001.

**FIGURE 4:**
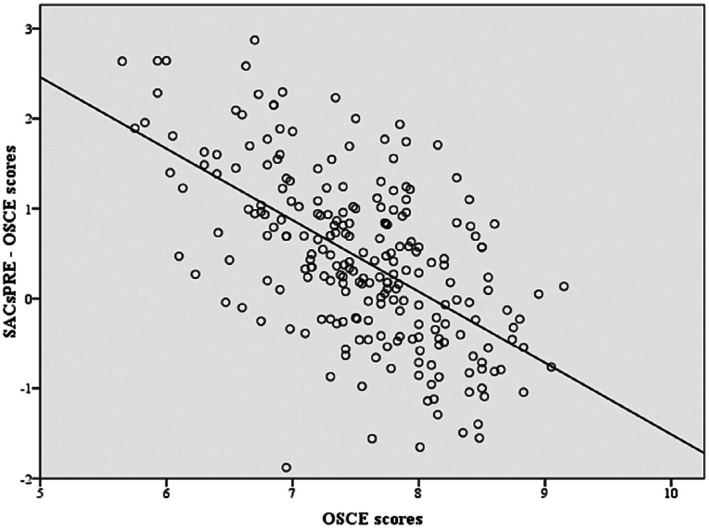
Scatter plots of differences in student self‐assessment pre‐OSCE competence evaluation and OSCE competence evaluation.

**FIGURE 5:**
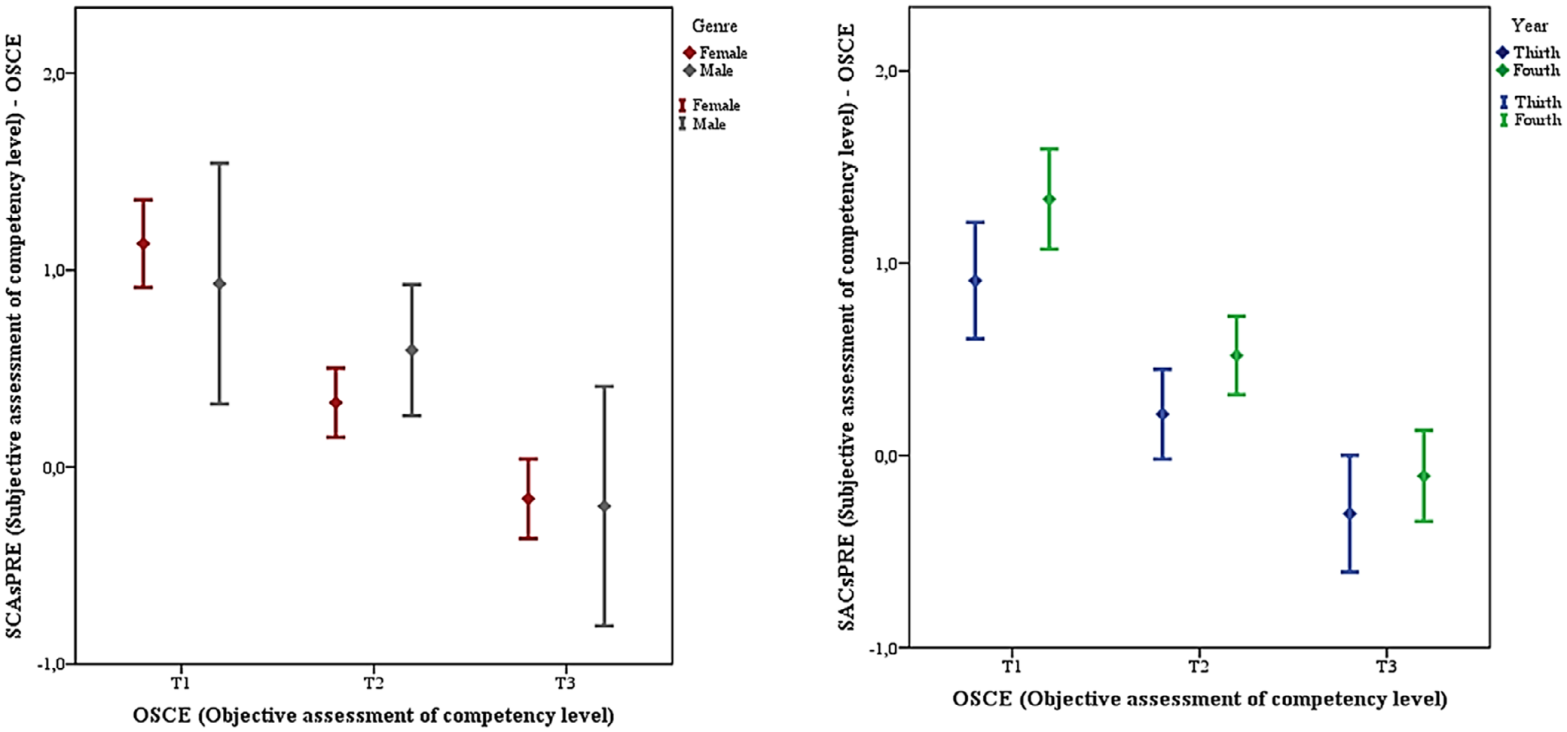
Tertile analysis difference between objective assessment (OSCE) and self‐perception of competence level, by genre and year of university studies.

The linear regression analysis showed a significant difference between the evaluation of competence levels measured through a self‐assessment tool (total adjusted pretest SACs less OSCE) and a standardised tool (OSCE examination) (beta coefficient = −0.794; 95% CI = [−0.94, −0.65]; *p* < 0.001) (Figure 4). With more detail, and after analysing the sample by tertiles according to the objective results of the objective competency level (OSCE result), significant differences were observed regarding the self‐perception of competency level (pretest SACS) (*F* = 47.04; *p* < 0.001). This was a large effect size for tertile one with a Cohen's *d* of 1.21, a medium effect for tertile two with a Cohen's *d* of 0.53, and a small effect for tertile three, with a Cohen's *d* of 0.21. Differences by gender and year of study can be graphically appreciated in Figure 5.

## 5 Discussion

The study revealed significant insights into the influence of objective structured clinical examinations (OSCEs) on nursing students' self‐assessment of practical competencies. While overall self‐assessment scores (SACs) remained stable, changes were observed across specific competency areas. Scores decreased in technical and procedural domains such as documentation, technical skills and communication, while they increased in clinical assessment, health promotion and teamwork. These results suggest that OSCEs tend to reinforce applied and clinical reasoning competencies more than interpersonal or person‐centered skills, which often require deeper experience and personal interaction. Additionally, a moderate correlation between OSCE scores and posttest SACs scores indicates that students adjust their self‐assessment after undergoing objective evaluation. However, the weak correlation with pretest SACs scores and the differences between the objective competences results (OSCE scores) and the self‐perceived competence results (pretest SACs scores) adjusted by tertiles suggests that many students may have initially overestimated their abilities, reflecting the Dunning–Kruger effect. This cognitive bias highlights a gap in self‐awareness, emphasising the need for targeted feedback and reflective practices to foster accurate self‐evaluation and improve nursing education outcomes.

Fourth‐year students reported significantly higher self‐assessment scores compared to third‐year students, both before and after the OSCE. This finding aligns with previous studies (Kajander‐Unkuri et al. 2020) and may be explained by the increased number of clinical placements, greater exposure to simulation and more advanced training received by students in later academic years. These factors lead to a more comprehensive education. Consequently, students report increased self‐efficacy, greater perceived success and greater competence.

Consistent with prior research (Høegh‐Larsen et al. 2023), no significant differences were found in overall pre‐ and posttest SACs scores by gender or course. However, fourth‐year students showed slight improvements in self‐perception, while third‐year students exhibited a slight decrease, likely due to increased stress from encountering their first OSCE. This can be explained because fourth‐year students, having completed previous OSCEs and accessed more resources, may experience lower anxiety and perform better. Besides, fourth‐year students also have access to a greater number of clinical rotations and a range of resources and didactic tools, which may contribute to their ability to cope with the OSCE more effectively. Additionally, incorporating formative OSCEs with feedback in earlier courses could enhance learning significantly and reduce OSCE‐related anxiety.

When analysing competence clusters, the OSCE led to decreased self‐assessment in *Documentation, Technical and skills* and *Communication*, but significant improvements in *Clinical Assessment, Ethical decision making, Health promotion* and *Education interventions* and *Teamworking*. Overall, these findings show that students significantly improved their assessment in aspects related to the routine application of theory, whereas a minor improvement was observed in the competencies related to critical thinking, reinforcing the notion that OSCEs strengthen applied competencies more than interpersonal or person‐centered skills. This could be because these skills are the most challenging since they demand practical experience rather than solely theoretical application. This aligns with previous studies highlighting the challenges nursing students face in delivering person‐centered care, often due to limited interaction with patients and insufficient training in these concepts (Kavuran et al. 2024; Kim and Kim 2023), suggesting concrete areas for curricular improvement.

Regarding gender, although total SACs scores were similar, differences were found in specific competencies. Women tended to rate themselves higher in soft skills, such as ethical reasoning and teamwork, while men scored higher in technical skills. This finding are in line with existing literature, which suggests that female students may underestimate their performance due to lower self‐confidence (Heath et al. 2022) and higher anxiety during an OSCE (Stojanovic et al. 2018), whereas male students tend to overestimate performance (Madrazo et al. 2018). In general, male students exhibit less academic effort and lower academic achievement than female students do (Workman and Heyder 2020).

This finding also suggests that students with lower OSCE scores (tertile one) tend to overestimate their competencies before the exam, as highlighted in the results due to the large effect size adjusted by tertiles, similar to the evidence results in medical (Knof et al. 2024) and nursing students (Høegh‐Larsen et al. 2023). This overestimation, described by Kruger and Dunning (2000), arises because less competent individuals lack the metacognitive skills to recognise their limitations. Paradoxically, in the present study, improving the OSCE scores helped them recognise the limitations of their abilities. Results are also consistent with evidence indicating that once individuals become aware of what they do not know or cannot do, they are able to assess themselves more accurately (Atir et al. 2024; Fekonja et al. 2017; Høegh‐Larsen et al. 2023; Knof et al. 2024). Overall, it is essential that students as well as health care professionals have an objective view of their own performance to stay up‐to‐date and allow them to reinforce good practices and correct their actions as scientific evidence on clinical practice evolves.

Notably, the simulated environment has limitations caused by the artificial setting and observation‐based assessment, which is not free from the subjectivity of evaluators (Bullard et al. 2019; Newman et al. 2021). Furthermore, students might know their grades, but they might not know what their strengths and weaknesses are from the assessment (Piper et al. 2019; Yan 2020). Incorporating structured lecturer feedback into OSCE rubrics could address this issue (Wong et al. 2022). Furthermore, the use of Entrusted Professional Activities (EPAs) in clinical practice could enhance feedback by clarifying both the level of autonomy and the areas needing support (Lau et al. 2020; Nguyen‐Tri et al. 2024). In this way, EPAs are defined as “tasks or responsibilities that faculty entrust to a trainee to execute, unsupervised, once he or she has obtained adequate competence” (Pool et al. 2023). EPAs have been proposed to bridge the gap between education and practice by facilitating the translation of competencies into clinical practice.

Ultimately, aligning self‐assessment with objective evaluations like the OSCE is essential to ensure that students correctly identify learning needs. This alignment as defined in the literature not only promotes professional growth but also enhances patient safety (Høegh‐Larsen et al. 2023).

It is also important to note that the positive results obtained with the OSCE, both in this study and in previous research carried out in Spain and internationally, have led to its stable and definitive incorporation as a standard assessment method in nursing education (Høegh‐Larsen et al. 2023; Kajander‐Unkuri et al. 2020). The OSCE's structured, objective and competency‐based approach has demonstrated its capacity to reliably evaluate practical clinical skills, making it a benchmark for the assessment of nursing students, serving both summative and formative purposes. This consolidated implementation reflects the OSCE's positive impact on students' clinical competence, self‐awareness and readiness for professional practice, reinforcing its essential role in the quality assurance of nursing education in Spain and across Europe (Høegh‐Larsen et al. 2023; Kajander‐Unkuri et al. 2020).

### 5.1 Strengths and Limitations

In this study, we explored the influence of an OSCE on the self‐assessment of general practical nursing through specific competences. This approach improves accuracy, as self‐assessment tends to yield more precise evaluations. Moreover, focusing on specific competencies encourages deeper learning and increases problem‐solving skills in nursing students (Kajander‐Unkuri et al. 2020).

Another strength is the design of the study, which included assessments both immediately before and after the OSCE. This minimised the risk of external contamination and ensured that the measured changes were closely related to the intervention. Furthermore, the intervention was part of a mandatory academic activity, which reduced follow‐up losses while participation in the study remained voluntary.

However, this research has some limitations. First, this quasi‐experimental pre‐post study did not include a control group, which limits the ability to establish causal relationships between self‐assessment using the SACs and OSCE performance. Without a control group, it is not possible to determine to what extent the observed improvements are due to the intervention itself or to other external factors. However, this type of design allows the assessment of changes within the same individuals before and after the intervention, offering valuable insight into learning outcomes. Quasi‐experimental pre‐post designs are commonly used in educational research due to ethical and logistical challenges associated with randomization and the inclusion of control groups. Nevertheless, future research should consider incorporating a control group to enhance internal validity and further clarify the impact of the intervention (Pardavila‐Belio et al. 2019). Second, the study was conducted at a single private university in Spain, which may limit the generalizability of the findings to other educational contexts. As such, the results of this study may not be directly transferable to public institutions or to settings in other countries. Nevertheless, previous studies have reported similar findings in different contexts (Ha and Lim 2023; Thabet et al. 2021) which lends support and consistency to the results presented here. Third, although the Self‐Assessment Competency Scale (SACs) demonstrated good internal consistency in this study, research validating the scale is scarce. The scale is based on the epistemological foundation outlined by the Ministry of Education's ministerial order, which provides a solid theoretical basis. The instrument was created specifically for nursing students and tested in the context of an objective structured clinical examination within the nursing degree program (Rodríguez‐Higuera 2014). However, the tool has not yet undergone extensive validation across different languages, populations or educational contexts, which may affect the interpretability and generalizability of the self‐assessment results. Therefore, while the SACs scale offers a theoretically grounded approach to competency self‐assessment, caution is warranted when drawing conclusions based solely on these scores. A fourth limitation concerns the time gap between data collection (2019) and the current publication, although this does not substantially undermine the relevance of the findings. While the OSCE remains a relevant, standard evaluation tool and widely used in nursing education worldwide, possible changes in curricula or institutional policies during this period could affect the direct applicability of the results. Nevertheless, the results provide valuable insights into students' self‐assessment and competency perception, central constructs of this study and essential elements in healthcare education. Future research could explore how these perceptions evolve over time in response to curricular updates and educational innovations. Finally, the potential for evaluator bias should be acknowledged. Although each station was assessed by a single external examiner unfamiliar with the students, some degree of subjectivity is inherent in observational methods. Specific training was provided to ensure consistency and adherence to the OSCE criteria, but future research could benefit from using multiple assessors and inter‐rater reliability measures.

### 5.2 Implications for Nursing Education

The findings of this study have several implications for nursing education. Identifying the influence of OSCEs on self‐assessment encourages nursing students to engage in deeper self‐reflection. Through structured feedback from OSCEs, students will become more aware of their strengths and areas for improvement, fostering a habit of continuous self‐assessment and personal growth (Hernández Rivero et al. 2021). In addition, educators can help students better understand the gap between their perceived and actual clinical skills. This awareness can drive targeted learning, leading to improved clinical competence and preparedness for real‐world scenarios. Finally, insights gained from self‐assessment in OSCEs can be used to develop personalised learning plans for students. Educators can tailor instructional methods and remediation strategies to address specific weaknesses, thereby ensuring that students receive the support they need to succeed (Buring et al. 2022; Heath et al. 2022).

## 6 Conclusion

This study aimed to explore the impact of objective structured clinical examinations (OSCEs) on nursing students' self‐assessment of their general practical nursing competencies. The results suggest that OSCEs may significantly influence students' self‐perception of their practical abilities. A weak correlation was found between OSCE performance and pretest SACs scores, but a moderate correlation was found between OSCE performance and posttest SACs scores, indicating that self‐assessment becomes more aligned with actual performance after the OSCE. Additionally, the gap between students' self‐assessment and OSCE scores widened as OSCE ratings decreased, suggesting that students with lower OSCE scores tend to overestimate their competence. This finding reflects the presence of the Dunning–Kruger effect in the self‐assessment practices of nursing students.

These findings highlight both the accuracy and potential biases in self‐assessment, revealing that students' evaluations of their competencies can be influenced by their actual performance. Recognising these dynamics is crucial for improving educational strategies that promote realistic self‐evaluation and thus for ultimately enhancing nursing students' ability to accurately gauge and improve their skills. These findings should inform the design of future studies for prospective researchers to follow and conduct.

The data that support the findings of this study are available from the corresponding author upon reasonable request. The data are not publicly available due to privacy and ethical restrictions regarding the nursing students' information.

## Author Contributions

**Virginia La Rosa‐Salas:** conceptualization, methodology, investigation, resources, writing – original draft, supervision and project administration. **Carmen Fernández‐Panadero:** methodology, software, data curation. **Miriam Pereira‐Sánchez:** writing – review and editing. **Miren Idoia Pardavila‐Belio:** writing – review and editing. **Marta Lizarbe‐Chocarro:** methodology, software, validation, formal analysis, data curation, writing – original draft, visualisation.

## Funding

The authors have nothing to report.

## Disclosure

Number of references: In view of the variety of disciplines involved (self‐assessment, structured objective clinical assessment, Dunning‐Kruger effect) and the paucity of primary bibliography, we have included more than 25 references. The latter has necessitated the inclusion of bibliographies from a maximum of 7 years old, although the majority of references are less than 5 years old.

## Conflicts of Interest

The authors declare no conflicts of interest.

## Data Availability

The data that support the findings of this study are available from the corresponding author upon reasonable request.
